# Infections, autoimmunity and immunodeficiencies are the leading etiologies of non-cystic fibrosis bronchiectasis in adults from the southwest of Colombia.

**DOI:** 10.7705/biomedica.7500

**Published:** 2024-12-23

**Authors:** Andrés F. Zea-Vera, Carlos Andrés Rodríguez, Sebastián Giraldo, Mario Alejandro Chacón, Luis Fernando Guerrero, Ricardo Mosquera, Raúl Andrés Vallejo, Fabio Samir Vargas, María Andrea García, María A. Rengifo, Anilza Bonelo, Maximiliano Parra

**Affiliations:** 1 Departamento de Microbiología, Facultad de Salud, Universidad del Valle, Cali, Colombia Universidad del Valle Universidad del Valle Cali Colombia; 2 Laboratory of Clinical Immunology and Microbiology, Division of Intramural Research, National Institute of Allergy and Infectious Diseases, National Institutes of Health, Bethesda, MD, USA National Institutes of Health National Institute of Allergy and Infectious Diseases USA; 3 Departamento de Medicina Interna, Facultad de Salud, Universidad del Valle, Cali, Colombia Universidad del Valle Universidad del Valle Cali Colombia; 4 Servicio de Neumología, Clínica Neumológica del Pacífico, Cali, Colombia Clínica Neumológica del Pacífico Clínica Neumológica del Pacífico Cali Colombia; 5 Division of Hematology and Medical Oncology, Mayo Clinic, Phoenix, AZ, USA Mayo Clinic Mayo Clinic AZ USA

**Keywords:** Bronchiectasis, tomography, thorax, agammaglobulinemia, tuberculosis, alpha- 1-antitrypsin, pulmonary disease, chronic obstructive, lung, adults., bronquiectasia, tomografía, tórax, agammaglobulinemia, tuberculosis, alfa-1-antitripsina, enfermedad pulmonar obstructiva crónica, pulmón, adultos.

## Abstract

**Introduction.:**

Non-cystic fibrosis bronchiectasis is a complex medical condition with multiple etiologies, characterized by chronic productive cough and radiologic evidence of airway lumen dilation and wall thickening. Associated exacerbations and declining lung function contribute to increasing disability and mortality. There are no data about the prevalence of non-cystic fibrosis bronchiectasis etiologies in the Colombian population.

**Objective.:**

To investigate non-cystic fibrosis bronchiectasis etiology and clinical characteristics in adults evaluated in the southwest of Colombia.

**Materials and methods.:**

We conducted a cross-sectional, non-interventional study. Subjects diagnosed with non-cystic fibrosis bronchiectasis were referred to by their healthcare providers and then enrolled between October 2018 and April 2021. Medical records and radiological studies were evaluated. Participants underwent laboratory tests, including complete blood count, serum immunoglobulin levels, and, in some cases, additional tests.

**Results.:**

We included 161 subjects. The average age was 50 years old, and 59% were females. Bronchiectasis etiology was identified in 84.6% of the cases. Post- infectious (34.6%) and immune disorders (25.3%), represented by autoimmunity (13.6%) and immunodeficiency (11.7%), were the leading causes. Gender differences were noted in autoimmune (females: 18.8% versus males: 6.1%, p = 0.021) and immunodeficiency-related bronchiectasis (males: 21.2% versus females 5.2%, p = 0.002). Immunodeficiencies-associated bronchiectases were more frequent in subjects under 50 years of age, while chronic obstructive pulmonary disease-associated bronchiectases were common in subjects over 50 years of age.

**Discussion.:**

The etiologies of non-cystic fibrosis bronchiectasis in Colombia are diverse, exhibiting notable differences from other global regions. Serum immunoglobulin levels and clinical immunologist consultation should be prioritized in diagnosing patients with unclear bronchiectasis etiology, particularly those with recurrent sinopulmonary infections.

## Resumen

**Introducción.:**

Las bronquiectasias no relacionadas con la fibrosis quística son una condición médica compleja con múltiples causas etiológicas, caracterizada por tos crónica productiva y evidencia radiológica de dilatación de la luz de las vías respiratorias y engrosamiento de de la pared bronquial. Las exacerbaciones asociadas y el deterioro de la función pulmonar contribuyen a un aumento de la discapacidad y la mortalidad. No existen datos sobre la prevalencia de las causas etiológicas de las bronquiectasias no relacionadas con la fibrosis quística en la población colombiana.

**Objetivo.:**

Investigar la etiología y las características clínicas de las bronquiectasias no relacionadas con la fibrosis quística en adultos del suroccidente colombiano.

**Materiales y métodos.:**

Se desarrolló un estudio transversal, no intervencionista. Se incluyeron sujetos con diagnóstico de bronquiectasias no relacionadas con la fibrosis quística entre octubre de 2018 y abril de 2021, remitidos por sus médicos tratantes. Se evaluaron su historia clínica y los estudios radiológicos. A todos los participantes se les practicaron exámenes de laboratorio que incluyeron hemograma y niveles séricos de inmunoglobulinas, entre otros estudios complementarios.

**Resultados.:**

Se incluyeron 161 sujetos. La mediana de edad fue de 50 años y el 59 % eran mujeres. La causa de las bronquiectasias se identificó en el 84,6 % de los casos. Las principales causas fueron de carácter postinfeccioso (34,6 %) o trastornos inmunológicos (25,3 %), representados por autoinmunidad (13,6 %) e inmunodeficiencia (11,7 %). Se observaron diferencias de sexo en las bronquiectasias atribuidas a autoinmunidad (mujeres: 18,8 % *versus* hombres: 6,1 %, p = 0,021) y a inmunodeficiencias (hombres: 21,2 % *versus* mujeres: 5,2 %, p = 0,002). Estas últimas fueron más prevalentes en sujetos menores de 50 años, mientras que las bronquiectasias asociadas con la enfermedad obstructiva crónica (EPOC) fueron comunes en sujetos mayores de 50 años.

**Discusión.:**

Las causas etiológicas de las bronquiectasias no relacionadas con la fibrosis quística en Colombia son diversas y presentan diferencias notables en comparación con otras regiones del mundo. Los valores de las inmunoglobulinas séricas y la evaluación por parte de un inmunólogo clínico deben priorizarse en el estudio diagnóstico de los pacientes con etiología poco clara, particularmente en aquellos con infecciones sinopulmonares recurrentes.

Non-cystic fibrosis bronchiectasis represents a medical challenge due to its heterogeneity and complexity. It is a chronic lung condition characterized by a vicious cycle of infection and inflammation with an abnormal, permanent, and irreversible dilatation of the bronchi ^1^. Airway dilatation causes alterations in mucociliary function with improper elimination of mucus and microorganisms, increasing susceptibility to colonization and infection by different pathogenic microorganisms. Non-cystic fibrosis bronchiectasis creates a positive feedback loop consisting of chronic inflammation, dysregulation of the immune response, and airway injury that eventually leads to decreased lung function, respiratory failure, and potentially death ^1^^,^^2^. Clinical presentation includes chronic productive cough, recurrent respiratory tract infections (exacerbations) and, in some cases, hemoptysis, fatigue, malaise, weight loss, and findings associated with abnormal thickening and dilatation of the bronchial wall visible on lung imaging ^3^^-^^5^.

The prevalence of bronchiectasis increases with age and, depending on the region analyzed, affects more women than men ^1^. However, the overall prevalence is changing rapidly, as reported in the United States and Europe, where it increased around 40% in the last decade ^6^. Currently, the estimated prevalence varies significantly between world regions, being 67, 362, 701, and 1,200 per 100,000 individuals in Germany, Spain, the United States, and China, respectively ^7^^-^^10^. This difference between regions may be due to many factors, such as diagnostics tools, environmental and sociodemographic variables, and the availability of a bronchiectasis registry ^1^. To the best of our knowledge, studies exploring the etiology of bronchiectasis are scarce in Latin America.

Several conditions, including respiratory infections (bacterial and mycobacterial), systemic autoimmunity, allergic bronchopulmonary aspergillosis, inborn errors of immunity, secondary immunodeficiencies, *ciliopathies* (for example, primary ciliary dyskinesia) and other diseases, such as gastroesophageal reflux disease, asthma, and chronic obstructive pulmonary disease are the leading causes of bronchiectasis ^5^.

The etiological characterization of non-cystic fibrosis bronchiectasis (epidemiology, clinical presentation, and severity) is essential to make a timely diagnosis and initiate an appropriate treatment. Thus, physicians can impact the quality of life and prognosis of patients ^1^^,^^3^^,^^4^. Although there are different cohorts of patients published around the world, there is no national data in Colombia ^6^^,^^8^^-^^14^.

This study is the first to characterize prospectively the etiology, demographic, and clinical features of subjects presenting with non-cystic fibrosis bronchiectasis in the southwest of Colombia.

## Materials and methods

### Study subjects

This study is cross-sectional and non-interventional. We recruited participants diagnosed with non-cystic fibrosis bronchiectasis in Cali, southwest Colombia, at various clinics, medical offices, and at *Hospital Universitario del Valle* between October 2018 and April 2021.

Participants were referred for enrollment to our center at *Universidad del Valle* for further evaluation by physicians (pulmonologists, internists, allergists, or primary care providers) taking care of these patients. All patients had at least one normal sweat test during their lifespan to rule out cystic fibrosis. Following an interview with a physician, the clinical records of participants were evaluated to determine suitability for inclusion in the study. Once included, an additional evaluation was performed by an internist and a clinical immunologist trained in lung diseases. A sputum sample and 10 ml of peripheral blood were collected for immunological and microbiological testing.

The inclusion criteria were subjects aged between 13 and 66 years, diagnosed with non-cystic fibrosis bronchiectasis according to the first recommendations published by the British Thoracic Society in 2010 ^15^, and the ability to give informed consent. Individuals with bronchiectasis due to cystic fibrosis, traction bronchiectasis associated with interstitial lung disease or another respiratory disorder, acquired immune defects or secondary immunodeficiencies (chronic myeloid leukemia, multiple myeloma, HIV infection, or immunosuppression secondary to drugs) were excluded from the study.

### Data collection

For each participant, data was collected through an interview guided by a recruitment form designed by the researchers and approved by the institutional review board. Medical records and previous imaging studies were reviewed with the participants. We performed an interview and physical examination to determine age, sex, body mass index, smoking status, biomass exposure, and medical history. We extracted the characteristics of respiratory tract infections from medical records, modified Medical Research Council (mMRC) dyspnea scale, spirometry variables, lung lobe involvement, pattern of bronchiectasis, and previous sputum or bronchoalveolar lavage cultures.

The underlying cause of non-cystic fibrosis bronchiectasis for each participant was reported by the treating clinician (pulmonologist or internist). The principal investigator corroborated or defined the cause of non-cystic fibrosis bronchiectasis in cases where the diagnosis was uncertain or discordant between two or more physicians.

### Diagnostic tools for assessment

Laboratory testing was done and supported by the study, according to the recommendations of the British Thoracic Society guideline for bronchiectasis in adults (2018) ^16^. A complete blood count, serum immunoglobulin levels quantification (total IgA, IgM, IgG, and IgE), and gene-specific tests (*SERPINA1*) were conducted in all participants to investigate for inborn errors of immunity, secondary immunodeficiencies, or specific genetic alterations as alfa-1-antitrypsin deficiency.

Based on these test results and clinical records, we carried out the following studies:

- Cases with low serum immunoglobulin levels were confirmed with a new sample and absolute quantification of IgG subclasses (IgG1, IgG2, IgG3, and IgG4).
- Hypergammaglobulinemia was tested with serum protein electrophoresis and immune fixation to exclude monoclonal gammopathies.
- According to total IgE values, aspergillus-specific IgE or skin prick test were performed with participants’ samples to investigate for allergic bronchopulmonary aspergillosis.
- Antecedents of pneumococcal infection or high clinical suspicion of antibody deficiencies were tested for IgG anti-pneumococcal polysaccharides.
- A sweat test for cystic fibrosis was performed for patients without available medical records.
- Alpha-1-antitrypsin deficiency was evaluated using SERPINA1 genotyping test (polymerase chain reaction amplification and hybridization with probes using Luminex xMAP™ for the 14 allelic variants associated with A1 AT) in all the subjects.
- Molecular testing for primary ciliary dyskinesia was conducted on patients with supporting clinical features.
- We used sputum cultures or GeneXpert™ kit approach for patients presenting with a productive cough at enrollment; and
- HIV-ELISA was offered to all patients.

Immunological variables such as lymphocyte subsets or flow cytometry tests were obtained from the medical records of some participants. Furthermore, the test results provided by the participants involved spirometry and chest computed tomography findings (including radiological evaluation and additional pulmonologist observations).

Lung function (spirometry) was defined as described previously ^17^. Briefly, normal spirometry was FEV_1_ and FVC > 80% of predicted value and FEV_1_ /FVC ≥ 0.7; airflow restriction FEV_1_ or FVC < 80% and FEV_1_ /FVC ≥ 0.7; airflow obstruction FEV_1_ /FVC < 0.70 and mixed pattern as FEV_1_ or FVC < 80% and FEV_1_ /FVC ≤ 0.7.

### Definitions of etiologies of bronchiectasis

Definitions were established according to the literature ^11^ as follows.

Diagnosis of post-infectious bronchiectasis was made if the participant reported a history of symptoms due to bronchiectasis after a severe respiratory infection, such as pneumonia, tuberculosis sequelae, or active infection, according to clinical judgment and regardless of the latency between the event and the symptoms of bronchiectasis.

Bronchiectasis associated with chronic obstructive pulmonary disease was diagnosed with smoking history of at least ten pack-years with airflow obstruction (FEV/FVC ratio < 0.7) according to the Global Initiative for Chronic Obstructive Lung Disease ^18^.

Bronchiectasis associated with asthma was diagnosed in subjects who did not present with post-infectious bronchiectasis, blood tests with standard or negative results, and severe asthma history according to the Global Initiative for Asthma guidelines ^19^.

Diagnosis of autoimmunity-associated bronchiectasis was made in the presence of bronchiectasis and connective tissue disease, including systemic lupus erythematosus, rheumatoid arthritis, Sjogren syndrome, dermatomyositis, mixed connective tissue disease, or systemic sclerosis.

Allergic bronchopulmonary aspergillosis was diagnosed with:

- Serum IgE greater than 1,000 IU/ml and *Aspergillus fumigatus* precipitins greater than 10 IU/ml or
- Specific IgE against *Aspergillus* spp. plus sputum and/or peripheral blood eosinophilia (500 cells/µl) and central bronchiectasis ^20^.

Inborn errors of immunity were diagnosed when the participant met the European Society for Immunodeficiencies criteria and was classified according to the International Union of Immunological Societies ^21^. When participants with test-confirmed immunodeficiency did not meet the criteria for inborn errors of immunity and were associated with immune-mediated diseases, they were classified with a secondary immunodeficiency.

Primary ciliary dyskinesia diagnosis was made through molecular testing or evaluation of ciliary structure and function in subjects with supporting clinical features suggested by the European Respiratory Society Working Group guidelines ^22^.

Gastroesophageal reflux associated with bronchiectasis was diagnosed in participants presenting with reflux symptoms without another underlying etiology. This condition was defined according to National Institute for Health and Care Excellence clinical guidelines ^23^.

Other less common etiologies of bronchiectasis were evaluated according to clinical histories and laboratory findings and were classified as miscellaneous. Subjects who did not meet the diagnostic criteria for the proposed etiologies were categorized as having idiopathic bronchiectasis.

### Microbiological evaluation

All participants presenting with a productive cough at enrollment were offered sputum direct analysis (Gram and Ziehl-Neelsen stain) and microbiological cultures (bacteria and mycobacteria). Culture results and other microbiological findings-preceding enrollment-were included for analysis.

### Bronchiectasis severity evaluation

The severity of bronchiectasis was evaluated according to the Bronchiectasis Severity Index (BSI) ^8^. Briefly, participants were classified into mild (BSI = 0-4), moderate (BSI = 5-8), and severe (BSI > 9) disease ^24^.

### Statistical analysis

Continuous data were presented as median values with 25^th^ to 75^th^ interquartile ranges. Categorical data were presented as absolute numbers and percentages. Data visualization was performed using R, version 4.1.0.

### Ethics

The study was approved by the Institutional Committee of Human Ethical Review of the Universidad del Valle, approval number 010-019R, and by the Institutional Review Board of the Hospital Universitario del Valle, approval number 050-2019. The study was conducted following the principles of the Declaration of Helsinki.

### Consent to participate

At enrollment, written informed consent was obtained from all participants. In the case of minors (individuals under 18), the participant and the legal guardian provided assent and consent. All participants consented to the publication of results.

## Results

We included 161 patients in the study from 2018 to 2021. Their median age was 50 years (IQR = 35 - 58), and women were predominant (n = 95; 59%). The median body mass index was 22.2 (IQR = 19.1 - 25.5), and 81 patients (50.3%) reported never to smoke.

Additionally, 38 patients (23.6%) had a history of otitis or sinusitis, 45 patients (28%) informed having recurrent pneumonia, and 41 patients (25.5%) had pulmonary tuberculosis. At enrollment, 35 patients (21.7%) were hospitalized.

Hemoptysis was reported by only 15 patients (9.3%), while 11 patients (6.8%) presented with pulmonary hypertension. The median modified Medical Research Council dyspnea scale score was one (IQR = 0-2).

Among the 126 cases with available information, the median delay in bronchiectasis diagnosis was six years (IQR = 2-16). Detailed demographic and clinical information of the patients is presented in table 1.

**Table 1.** Sociodemographic and clinical data of the study population by bronchiectasis etiology

Parameter	Data available (n)	All (N = 161)	Post-infectious (n = 56)	Idiopathic (n = 25)	Autoimmunity (n = 21)	Immunodeficiency (n = 19)	Asthma (n = 11)	COPD (n = 8)	PCD (n = 8)	Other (n = 13)
Age (years)*	161	50 (35 - 58)	50 (36.5 -58.2)	54 (42 - 63)	54 (47 - 57)	30 (18-46.5)	38 (35.5-49.5)	60 (58 - 63.3)	31 (26 - 43.5)	42 (36 - 57)
Diagnosis delay	126	6 (2-16)	5 (2-.13)	11 (2.5-24.5)	3 (1 - 6)	8 (5-17)	10.5 (5-20.2)	4 (0 - 25)	7 (7 - 34)	6 (2 - 20.5)
(years)* Gender [n (%)]	161		56	25	21	19	11	8	8	13
Male		66 (41)	22 (39.3)	11 (44)	4(19)	14 (73.7)	2 (18.2)	3 (37.5)	3 (37.5)	7 (53.8)
Female		95 (59)	34 (60.7)	14 (56)	17(81)	5 (26.3)	9 (81.8)	5 (62.5)	5 (62.5)	6 (46.2)
Body-mass index*	157	22.2 (19.1 - 25.5)	21.2 (18.7-24.4)	23.2 (20.4 - 26)	22.7 (21-25.6)	20.8 (18.2-26.7)	25.4 (22.7 - 27.5)	22.8 (20.9 - 24)	20.1 (18- 22.4)	21.4 (19.5-25.2)
Smoking [n (%)]	161		56	25	21	19	11	8	8	13
Never		81 (50.3)	23 (41.1)	12(48)	11 (52.4)	14(73.3)	8 (72.7)	2(25)	6(75)	5 (38.5)
Former		22 (13.6)	10 (17.9)	5(20)	1 (4.8)	2(10.5)	0	3 (37.5)	0	1 (7.7)
Current		17(10.6)	6(10.7)	4(16)	1 (4.8)	2(10.5)	1 (9.1)	2(25)	0	1 (7.7)
Passive		41 (25.5)	17(30.4)	4(16)	8(38.1)	1 (5.3)	2(18.2)	1 (12.5)	2(25)	6 (46.2)
Biomass exposure [n (%)]	161	58 (36)	26 (46.4)	9(36)	7 (33.3)	2(10.5)	2(18.2)	5 (62.5)	3 (37.5)	4 (30.8)
Lungs and infection			56	25	21	19	11	8	8	13
Otitis or rhinosinusitis	161	38 (23.6)	16(28.6)	5 (20)	1 (4.8)	5 (26.3)	3 (27.3)	0	5 (62.5)	3(23.1)
Recurrent pneumonia	161	45 (28)	13(23.2)	2(8)	4(19)	14 (73.7)	3 (27.3)	1 (12.5)	6(75)	2(15.4)
mMRC score*	161	1 (0-2)	2 (1-3)	1 (0-1)	1 (1-2)	1 (1-3)	1 (1-2)	2 (1-3)	1 (0-1)	1 (0-3)
FEV_1_ (%predicted)*	100	66 (50 - 82.3)	60 (51.5 - 74.5)	82 (69-91)	78.5 (65 - 86)	47 (36 - 60)	67 (58 - 77.8)	54.5 (48.5 -57)	47.5 (32.5 - 64)	46 (36 - 69)
FVC (%predicted)*	100	71 (54 - 83.5)	73 (56 - 84)	76 (68 - 85)	78.5 (62 - 89.8)	52 (39 - 79)	75.5 (66.8 - 83)	64 (62.3 - 68.5)	53.5 (39.5 - 62)	51 (43 - 66)
Lung lobes affected [n (%)]	158		56	25	20	17	11	8	8	13
One		28 (17.7)	14(25.9)	5(20)	3(14.3)	0	3(25)	2 (28.6)	1 (16.7)	1 (7.7)
Two		54 (34.2)	19 (35.2)	9 (36)	5 (23.8)	7 (43.8)	3(25)	2 (28.6)	2 (33.3)	6 (46.2)
Three		31 (19.6)	10 (18.5)	4(12)	4(19)	3 (18.8)	1 (8.3)	0	2 (33.3)	4 (30.8)
Four		20 (12.7)	9 (16.7)	1 (4)	3 (14.3)	2 (12.5)	2 (16.7)	2 (28.6)	0	0
Five		8(5.1)	0	4(16)	2 (9.5)	0	1 (8.3)	0	0	1 (7.7)
Six		17 (10.8)	2 (3.7)	2(8)	4(19)	4(25)	2 (16.7)	1 (14.3)	1 (16.7)	1 (7.7)
Bronchiectasis pattern [n (%)]	139		48	22	19	14	12	7	6	11
Cylindrical		99 (70.7)	30 (62.5)	17 (77.3)	14 (73.3)	7(50)	8 (66.7)	6 (85.7)	4 (66.7)	4 (36.4)
Cystic		33 (37.1)	12 (25)	3 (13.6)	4(21.1)	4 (28.6)	3 (16.7)	1 (14.3)	2 (33.3)	1 (9.1)
Varicose		8 (19.3)	6 (12.5)	2(9.1)	1 (5.3)	3 (21.4)	1 (8.3)	0	0	5 (45.5)
Microbiology [n (%)]	100		43	14	8	14	6	3	5	7
*Pseudomonas*		15 (15)	4 (9.3)	3 (21.4)	0	1 (7.1)	1 (16.7)	0	4(80)	2 (28.6)
*Aeruginosa Staphylococcus*		5(5)	0	3 (21.4)	1 (12.5)	1 (7.1)	0	0	0	0
*Aureus Haemophilus*		6(6)	1 (2.3)	1 (7.1)	1 (12.5)	2(14.3)	0	1 (33.3)	0	0
*Influenzae Enterobacteriaceae*		7(7)	5(11.6)	0	0	0	0	0	1 (20)	1 (14.3)
Severity of the disease -BSI score, n (%)	161									
Mild (0 - 4)		68 (42.2)	17 (30.4)	15 (60)	10 (45.5)	6 (31.6)	9(75)	2(25)	2 (28.6)	7 (53.8)
Moderate (5 - 8)		51 (31.7)	18 (32.1)	8 (32)	8 (36.4)	4 (21.1)	2 (16.7)	5 (62.5)	3 (42.9)	4 (30.8)
Severe (> 9)		42 (26)	21 (37.5)	2(8)	4 (18.2)	9 (47.4)	1 (8.3)	1 (12.5)	2 (28.6)	2 (15.4)

COPD: Chronic obstructive pulmonary disease; PCD: Primary ciliary dyskinesia; mMRC: modified Medical Research Council dyspnea scale; FEV1: Forced expiratory volume in the first second; FVC: Forced vital capacity; BSI: Bronchiectasis severity index

* Data are presented as absolute and relative (in parenthesis) frequencies.

### Etiology of non-cystic fibrosis bronchiectasis

The etiology of non-cystic fibrosis bronchiectasis was discerned in 84.5% of patients (table 2). Post-infectious causes were the most prevalent (34.8%), including 36 cases of bronchiectasis associated with tuberculosis, 9 of recurrent pneumonia, and 11 of other infections. Immune disorders, resulting from autoimmunity or immunodeficiency, represented the second most common etiology with 40 cases (24.8%). Autoimmunity constituted 13% of the cases, with prevalent autoimmune diseases including systemic lupus erythematosus (n = 8), Sjogren’s syndrome (n = 3), and rheumatoid arthritis (n = 6). Inborn errors of immunity and secondary immunodeficiencies played a substantial role as underlying causes of bronchiectasis, with 19 cases (11.8%).

**Table 2:** Etiology of non-cystic fibrosis bronchiectasis in the study population presented as absolute and relative frequencies (N = 161)

Etiology		n	(%)
Post-infectious		56	(34.8)
	Tuberculosis sequelae	31	(19.2)
	Active tuberculosis	10	(6.2)
	Recurrent pneumonia	9	(5.6)
	Other infections	11	(6.8)
Immune disorders		40	(24.8)
Autoimmunity		21	(13.0)
	Systemic lupus erythematosus	8	(5.0)
	Sjögren's syndrome	3	(1.9)
	Rheumatoid arthritis	6	(3.7)
	Dermatomyositis	1	(0.6)
	Mixed connective tissue disease	1	(0.6)
	Systemic sclerosis	1	(0.6)
	ANCA-positive vasculitis	1	(0.6)
Immunodeficiency		19	(11.8)
	Inborn errors of immunity	13	(8.0)
	Secondary immunodeficiency	6	(3.7)
Idiopathic		25	(15.5)
Asthma		11	(6.8)
Chronic obstructive pulmonary disease		8	(5.0)
Primary ciliary dyskinesia		8	(5.0)
	Confirmed	4	(2.5)
	High suspicion	4	(2.5)
Gastroesophageal reflux disease		5	(3.1)
Allergic bronchopulmonary aspergillosis		2	(1.2)
Miscellaneous		6	(3.7)

Among bronchiectasis cases associated with lung diseases, asthma accounted for 6.8%, and chronic obstructive pulmonary disease for 5%. Other identified causes of bronchiectasis included primary ciliary dyskinesia (5%), gastroesophageal reflux disease (3.1%), allergic bronchopulmonary aspergillosis (1.2%), and miscellaneous factors (3.7%). We did not identify a specific etiology in 25 subjects, so they were classified with idiopathic bronchiectasis.

**Supplementary figure 1.**
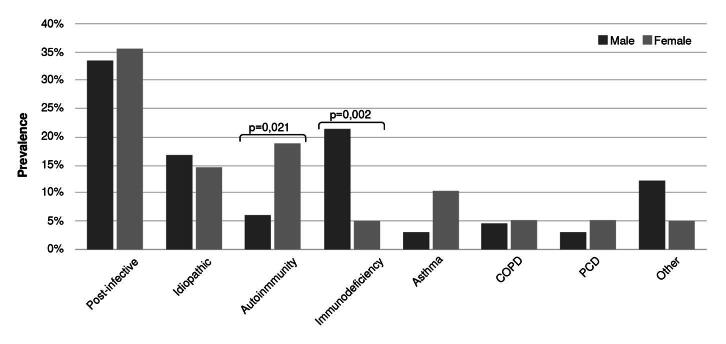
Etiology of bronchiectasis differentiated by sex COPD: Chronic obstructive pulmonary disease; PCD: Primary ciliary dyskinesia The “other” category include tetralogy of Fallot, diffuse interstitial lung disease, hypersensitivity pneumonitis, multiple myeloma, and ectrodactyly/ectodermal dysplasia-cleft.

A gender-based analysis revealed two etiologies with significantly different prevalence rates between male and female patients: Autoimmunity- related bronchiectasis was more frequent in females (6.1% versus 18.8%; p = 0.021), while immunodeficiency-related bronchiectasis was more common in males (21.2% versus 5.2%; p = 0.002) (Supplementary figure 1). Immunodeficiencies, particularly inborn errors of immunity, were significantly more prevalent in patients under 50 years, whereas chronic obstructive pulmonary disease associated with bronchiectasis was notably more frequent among those aged 75 or older.

Before enrollment, serum immunoglobulin levels had been tested in less than 15% of patients. After immunoglobulin evaluation (IgG, IgA, IgM, and IgE) in all participants, four patients were diagnosed with de novo predominantly antibody deficiencies: Two with common variable immunodeficiency and two with selective IgA deficiency. These results amounted to 13 patients (8%) with bronchiectasis attributable to inborn errors of immunity. Molecular evaluation in all participants did not reveal alpha-1- antitrypsin deficiency cases.

**Figure 1.**
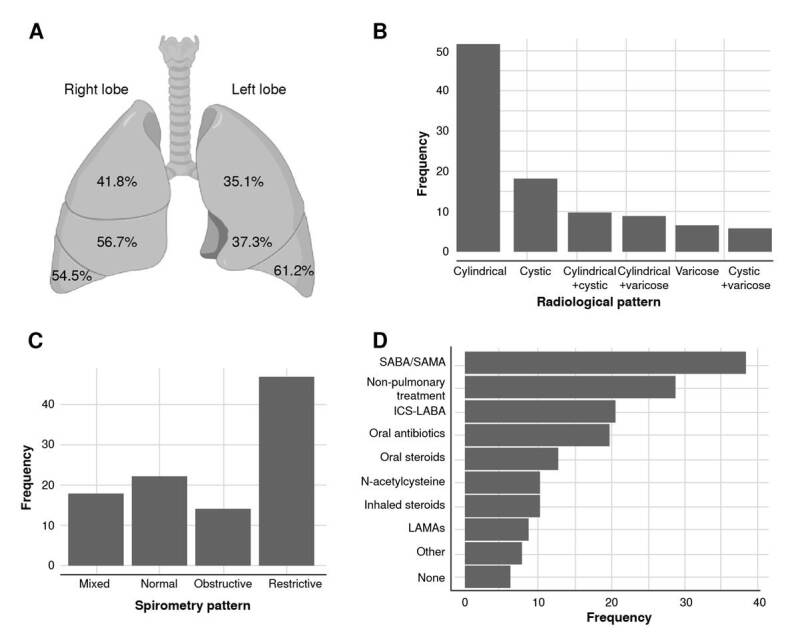
Lung involvement and pharmacological treatment in patients with non-cystic fibrosis bronchiectasis from southwestern Colombia. A) Distribution of bronchiectasis in lung lobes (the lingula was considered a lobe); B) Bronchiectasis pattern on computed tomography; C) Spirometry pattern at baseline (pre-bronchodilator); D) Prescribed treatment for the participants with bronchiectasis. All frequencies are expressed as percentages. SABA: Shortacting beta agonists; SAMA: short-acting muscarinic antagonists; ICS: Inhaled corticosteroids; LABA: Long-acting beta- agonists; LAMA: Long-acting muscarinic antagonists.

Concerning the distribution of bronchiectasis across pulmonary lobes, the right lung’s lobes were more frequently affected, with the lower lobes exhibiting a higher frequency of bronchiectasis compared to the upper lobes in both lungs (figure 1A). Radiological examination revealed cylindrical dilatation as the most prevalent finding (48.9%), followed by cystic (18%) and coexisting cylindrical and cystic bronchiectasis (14.4%) (figure 1B).

Spirometry indicated that most patients exhibited a restrictive pattern (60.4%), while a minority showed a mixed pattern (obstructive and restrictive) (7.7%) (figure 1C). The median percentage of predicted FEV, was 67 (IQR = 52 - 83), and the median percentage of predicted FVC_1_ was 72.5 (IQR = 54 - 83.5) (table 1). An association was observed between the spirometry and the most frequent bronchiectasis pattern (p value < 0.05), with restrictive and obstructive patterns linked to cylindrical bronchiectasis.

An analysis of the number of lobules affected per case revealed that 53 patients (34.4%) had two lobes affected, while 29 patients (18.8%) had only one lobe with dilatation. Other patients had more than two lobes affected, including 25 participants (16.2%) with more than four lobes with bronchiectasis (table 1). No significant association was found between the number of affected lobes and bronchiectasis pattern, spirometry pattern, FEV_1_, or FVC.

The most prevalent treatment modality was inhaled bronchodilators (37%). The second most common treatment involved a combination of inhaled corticosteroids and long-acting bronchodilators (17.9%), followed by oral antibiotics, steroids, and N-acetylcysteine. Treatment strategies were customized based on the specific etiology of bronchiectasis, including immunosuppressive medications for autoimmune-related cases and intravenous immunoglobulins for patients with predominantly antibody deficiencies. A small proportion of patients (17.3%) did not receive treatment at enrollment (figure 1D).

While sputum analysis was available for all patients, only 35 cases presented with a productive cough at enrollment. Of the 100 patients with available microbiology test results (records), 15% had Pseudomonas aeruginosa, and 5% had *Staphylococcus aureus*. Grouping participants according to bronchiectasis etiology (table 1) revealed an association between post-tuberculosis bronchiectasis and current smokers (p values < 0.05).

No statistical differences were observed for the remaining variables between the bronchiectasis groups.

## Discussion

To our knowledge, this is the first prospective study in Colombia presenting the etiologies of non-cystic fibrosis bronchiectasis in adults. We identified bronchiectasis causes in 84.5% of the cases. This finding contrasts with other observational studies conducted in South America (five studies in Brazil with 308 individuals), which found an etiology in only 62.7% of the cases. These Latin American studies align with pooled findings from 56 international studies describing the etiology in 55.2% of 8,608 participants ^25^. Low- idiopathic bronchiectasis etiology was achieved through a rigorous evaluation that enabled accurate etiological identification. However, it is important to acknowledge that bronchiectasis with unknown etiology remains a significant challenge in clinical practice without standardized criteria for its definition.

Among the etiologies identified in our study, the post-infectious cause was the most prevalent (34.8%), with tuberculosis being the predominant subcategory. A retrospective and etiological study conducted in Brazil with 77 participants with bronchiectasis found *Mycobacterium tuberculosis* infection as the primary cause (41.5%) ^26^. A systematic review compiled 29 studies (16 from Europe, 7 from Asia, 4 from South America, 1 from North America, and 1 from Africa) reporting post-tuberculosis bronchiectasis and highlighting a significantly higher prevalence in Asia compared to Europe (68.5% versus 53.7%) ^25^.

The World Health Organization “Global Tuberculosis Report -2020” estimated 9.9 million new tuberculosis cases worldwide, with an incidence of 127 cases per 100,000 inhabitants. The 30 high-burden tuberculosis countries accounted for 86% of all estimated new cases globally, most of which are in developing countries ^27^^,^^28^. In 2021, Colombia had an incidence rate of 25.9 per 100,000 inhabitants, classifying it as a tuberculosis-endemic country ^29^.

Generally, bronchiectasis due to pulmonary tuberculosis presents as sequelae resulting from parenchymal damage during active infection ^30^^,^^31^. Additionally, the association between previous infections and the development of bronchiectasis remains elusive in clinical practice due to the inherent difficulty in determining the severity of the prior infection, temporal association, and the lack of standardized criteria for defining post-infectious bronchiectasis. However, our findings indicate that post-infectious etiology (particularly post-tuberculosis) remains an important category in Colombia that should continue being investigated.

Autoimmune diseases represented the second most important cause (13%) of bronchiectasis etiology. Our study found similar findings to different European cohorts, where 1,258 subjects from six countries were evaluated; connective tissue diseases accounted for 10% of the cases ^11^. Some authors have suspected a high prevalence of bronchiectasis in ANCA-positive vasculitis patients ^32^. In contrast, others demonstrated that these structural alterations may be a common extra-articular manifestation in rheumatoid arthritis ^33^. In our cohort, we identified only one subject with ANCA-positive vasculitis who developed multiple bronchiectasis, and 29% of the subjects with autoimmunity had rheumatoid arthritis. The association between bronchiectasis development and autoimmunity must be the research focus of groups worldwide.

Primary and secondary immunodeficiencies are among the leading causes of non-cystic fibrosis bronchiectasis in Colombian adults. As previously reported by Gao *et al*., immunodeficiencies represent 5% of cases. Immunodeficiency prevalence ranges from low, as reported in Brazilian studies, (1.9%) to high, as documented in a North American study (17%) ^25^. The European Multicenter Bronchiectasis Audit and Research Collaboration (EMBARC) registry describes a high proportion of patients diagnosed with immunodeficiencies, making it the fourth identifiable cause after chronic obstructive pulmonary disease and asthma ^34^. Immune dysregulation and immunodeficiency can lead to severe infections with extensive inflammation that extend structural damage and results in the formation of bronchiectasis. In Colombia, immunodeficiencies are rare as elsewhere, underserved conditions that should be investigated in patients with bronchiectasis and recurrent infections when excluding other prevalent etiologies.

Inborn errors of immunity were a significant cause of bronchiectasis in our study. Since our series excluded subjects with a prior diagnosis of secondary immunodeficiencies, we observed a higher representation of primary immunodeficiencies. Bronchiectasis represents the most frequent non-infectious pulmonary complication of inborn errors of immunity. A French retrospective study that included 98 patients with bronchiectasis from 1984 to 2012 found that 20.4% had an inborn error of immunity ^35^. A cohort study in Colombia evaluating subjects with recurrent pneumonia and bronchiectasis reported a prevalence of primary immunodeficiencies of 10% ^36^. Studies conducted to date report prevalences ranging from 1% to 17% ^13^^,^^37^^,^^38^. As expected, the most representative group of inborn errors of immunity was predominantly antibody deficiencies (76.9%), highlighting the importance of absolute measurement of serum immunoglobulin levels. Identifying patients with these disorders is crucial to change the course of the disease through early initiation of specific treatments such as immunoglobulin replacement. Therefore, we suggest implementing this easily accessible and cost-effective diagnostic tool as a mandatory component in different diagnostic algorithms to enhance these patients’ identification, aiming to delay disease progression and underlying lung damage ^39^.

The fourth most prevalent etiology was chronic respiratory diseases such as chronic obstructive pulmonary disease and asthma. This finding contrasts with the multicenter study by Lonni *et al*. ^11^ and the EMBARC registry ^34^, where chronic obstructive pulmonary disease is the second identifiable cause.

In our context, these findings may be due to improved detection, diagnosis, and timely treatment, which could delay the onset of bronchiectasis. On the other hand, asthma as an underlying etiology has a prevalence like that reported in these studies, ranking between the third and fifth causes.

Among the less common causes of bronchiectasis, primary ciliary dyskinesia was a diagnostic challenge. This condition appears in 1 to 3% of all cases reported in different series ^12^^,^^13^^,^^34^. We observed a higher frequency of primary ciliary dyskinesia in our cohort than that documented in the literature (5%). Therefore, we advocate for genetic evaluation as a diagnostic tool to consider when assessing subjects with bronchiectasis, especially after discarding more common etiologies.

Research on bronchiectasis, encompassing its epidemiology, etiology, and clinical features across diverse geographical regions, holds crucial significance. A mounting body of evidence suggests a geographical dependence on various disease-related variables, as highlighted ^40^. Nonetheless, significant knowledge gaps persist regarding bronchiectasis, particularly in low- to middle-income countries such as Colombia.

The number of subjects evaluated was limited. Those with a previously recognized secondary immunodeficiency or older than 66 years were excluded because elderly people has been reported independently as a relevant cause for bronchiectasis. Enrollment and participants’ inclusion were also affected by COVID-19 lockdowns in Colombia, which were more severe between March and December 2020.

One strength of this study is the prospective enrollment of the participants, unlike most other published studies. The lack of universal electronic medical records in Colombia and difficulties accessing the healthcare system and medical records represented a challenge.

Despite recognizing the limitations of our study, including enrollment bias, restricted availability of complementary testing, and absence of national electronic medical records, among others, our research stands as a pioneering effort. It enhances our comprehension of bronchiectasis in Colombia and makes valuable contributions to the refinement of diagnosis and treatment protocols for the patients under evaluation.

Our study performed a systematic evaluation, including clinical records, radiographical findings, immune tests, molecular analysis, and other strategies to identify the causes of bronchiectasis. Prospective enrollment and immunological evaluation done by a clinical immunologist is our biggest strength.

This study represents the first of its kind in our country, fostering a foundation for future investigations into this intricate pulmonary pathology in Colombia.
